# Myxobacteria: Physiology and Regulation

**DOI:** 10.3390/microorganisms10040805

**Published:** 2022-04-12

**Authors:** David E. Whitworth

**Affiliations:** Institute of Biological, Environmental and Rural Sciences, Aberystwyth University, Aberystwyth SY23 3DD, UK; dew@aber.ac.uk

Myxobacteria are fascinating and important prokaryotes. Their genomes are exceptionally large, endowing them with a wide range of interesting behaviors, including (but certainly not limited to) multicellular fruiting body formation, gliding motility, social interactions, predation, and secondary metabolite production. Their ecological importance in major ecosystems is becoming established, and we are beginning to understand the evolutionary forces that have shaped their current phenotypes and behaviors. Novel species of myxobacteria are steadily being discovered, often producing unusual metabolites and enzymes. There is also significant biotechnological interest in these organisms for a wide range of potential applications. Molecular studies, ranging in subject from individual enzymes to entire ‘omes, continue to provide rich insights into myxobacterial biology.

This Special Issue brings together five research articles and three reviews, to provide a snapshot of current myxobacterial research in all its diversity. We are very grateful to the authors for submitting their work to the issue.

Myxobacteria are well-known for their multicellular behaviors which are often associated with morphological changes [1]. When starved of nutrients, coordinated motility of cells in the population results in the formation of ripple patterns and multicellular aggregates, which develop into fruiting bodies. Inside the fruiting bodies, rod-shaped cells sporulate and remain dormant as round myxospores until nutrients become available once more. Zhang, H. et al., have provided a review of morphological changes during sporulation and germination, focusing on changes in peptidoglycan synthesis and degradation during the sphere-to-rod transition. In contrast, Zhang, J. et al., investigated population-level morphology, providing a novel computational approach for combining information from multiple types of microscopy.

The gene regulatory network underpinning fruiting body formation in myxobacteria is highly complex and it has taken decades of molecular genetics experiments to achieve our current understanding of the network [2]. Whitworth and Zwarycz surveyed regulatory genes in myxobacterial genomes to assess the conservation of signalling genes, also providing insights into how evolution has molded contemporary myxobacterial regulatory networks. Their analysis extended to quorum signaling systems and the Car system, which regulates the light-dependent production of photoprotective carotenoids. In their review, Padmanabhan et al., provide a thorough and mechanistically detailed description of the Car system-a particularly well-understood gene-regulatory network system which has also provided paradigms for widely distributed protein families with diverse functions.

Myxobacterial predation has received increasing research attention in recent years [3]. In this Special Issue, two research articles address aspects of the evolution and ecology of predation. Nair and Velicer used co-evolution experiments to investigate the evolutionary relationships between myxobacteria and their prey bacteria. They found that myxobacterial predators promote long-term diversity within prey communities. Mayrhofer et al., investigated the trophic interactions between nematodes, myxobacteria and prey bacteria, observing that myxobacteria and nematodes affect each other’s predatory behavior towards their shared prey.

Myxobacterial predation involves the secretion of secondary metabolites encoded by biosynthetic gene clusters, many of which are known to have antibiotic activity. Ahearne et al., undertook a genomic analysis of selected myxobacterial strains to assess their taxonomy and determine their biosynthetic potential, demonstrating that that genome-led classification can help prioritize natural product discovery. In their review of myxobacterial genomics, Whitworth et al., also focused on genome analysis as an aid for taxonomic classification, and showed how genomic information has enabled the application of post-genomic technologies to diverse aspects of myxobacterial research, including secondary metabolite biosynthesis.
